# Ethical Issues in Bereavement Research with Minors: A Scoping Review

**DOI:** 10.3390/children9091400

**Published:** 2022-09-15

**Authors:** Athena E. S. Park, Karolina Krysinska, Karl Andriessen

**Affiliations:** Centre for Mental Health, Melbourne School of Population and Global Health, The University of Melbourne, Parkville, VIC 3010, Australia

**Keywords:** ethics, research ethics, ethical issues, bereavement, grief, children, adolescents, youth, scoping review

## Abstract

There are various ethical issues in bereavement research. Most of the literature focuses on ethical issues involving adult participants. However, it is conceivable that research with minors poses particular ethical challenges, and little is known of the ethical issues involved in bereavement research with minors. A scoping review adhering to the PRISMA-ScR guidelines was conducted to address this gap and to contribute to better research practices. Searches in Embase, Emcare, EBM Reviews, Medline, PsycINFO (all accessed via Ovid), CINAHL, Scopus, SSCI, and the journals Death Studies and OMEGA identified 40 relevant peer-reviewed articles, while 25 relevant theses/dissertations were identified through ProQuest Global. The main ethical concerns identified include informed consent, risk to participants, and privacy and confidentiality. Findings of this review may inform bereavement researchers when designing their studies and to ensure the safety of their participants. The findings can also be used in clarifying the decisions made to a research ethics board, thus contributing to the quality of the research in this field. Future reviews may examine how the ethical issues reported in this review are similar or different to those reported in research with minors in other fields and expand to include more experimental research.

## 1. Introduction

There exists an established body of literature on the topic of ethical issues that arise in bereavement research [1,2,3]. This is unsurprising, as bereavement is characterized as an emotionally fraught period of time for study participants, since they lost someone they shared a relationship with. As such, the nature of bereavement makes it even more crucial that bereavement research treats participants’ well-being with utmost concern. Cook [1] provided an overview of general research guidelines that pertain to human subjects and how they might apply to bereavement studies, on the basis that bereavement research “has unique aspects that should be acknowledged” [1]. This overview raised several questions and observations, including: the subject of inadvertent coercion due to recruitment through care organizations, whether the recently bereaved are mentally fit to give consent, and the balance between providing all the information available so that individuals can make an informed decision versus making the information comprehensible without losing its original meaning (e.g., the connotations between “not living” and “death”) [2]. Overall, Cook [1] argued that ethical issues in bereavement research go beyond ethics guidelines and gaining approval from an ethical review board, which are not adequate substitutes for the researcher’s own sensitivities. It is argued that ethical issues should be addressed with the same diligence as the other parts of research design, considering how intertwined scientific issues and ethical concerns are. Moreover, Cook [1] posits that bereavement researchers should be less reactionary in addressing ethical issues, but rather take the initiative by generating discussion within the field. This is meant to simultaneously acknowledge the generosity of participants in sharing their experiences, as well as ensuring efforts to best safeguard their well-being is continuously ongoing.

Parkes [3] examined the ethical issues of conducting bereavement research. The observations fell into three broad categories: “gaining access to bereaved people,” “obtaining informed consent,” and “preventing possible harm to respondents”. Parkes developed a guideline based on the ethical issues he observed, which also addresses many of the questions posed and observations made in the overview by Cook [1]. The guideline for bereavement research included: gaining approval from the appropriate ethical approval board; matching communication with the participants to their cognitive level, with provisions made if the participant is cognitively impaired; avoiding coercion of potential recruits; ensuring safeguards are in place to minimize harm to the participants; remaining an impartial figure as a researcher; clearly outlining the bounds of confidentiality; and the research having merit and a design appropriate to answering the question(s) posed. Parkes’ guidelines are broad and provide general advice. However, there is little consideration given towards bereavement research involving vulnerable populations, including children and adolescents. Further, having been published in 1995, the guideline naturally did not contemplate more contemporary research settings such as the use of technology in the context of bereavement research [4].

Since Cook [1] noted that ethical issues in bereavement research were a little-explored topic, there have been strides in the area with researchers exploring the influence of scientific issues on ethical concerns. Stroebe and colleagues [5] considered the design and methodological aspects of bereavement research and addressed the associated ethical issues to include when and how best to approach potential participants after the bereavement. The question of “how soon?” is one without a definitive answer, as evidenced by how time since bereavement varies widely between studies. As well, a range of research has been conducted with various participant groups. Rosenblatt [6] focused on issues inherent in qualitative interviews with bereaved families, Hyson and colleagues [7] on bereaved parents, Omerov and colleagues [8] on those bereaved by traumatic deaths (specifically, suicide), and Feigelman and colleagues [9] on parents bereaved by suicide, to name a few studies.

There is also research regarding the experiences/perspectives of bereavement research participants, and whether participating causes undue harm or distress. It is noted that ethics research boards were found to reject research proposals more often on topics deemed as being sensitive [10]. As such, researchers have experienced a pushback against research proposals regarding death and related topics [1,11,12]. Gatekeepers, such as caretakers (e.g., parents, legal guardians), professionals (e.g., social workers, counsellors/therapists, medical staff), educational staff, and research ethics review boards, can also be reluctant to allow access to study participants due to the research topic [2,13]. However, for all the worries by these third parties, the bereaved themselves, both adults and minors, see the merits of bereavement research [10,14]. Overall, they reported that any distress that arose during participation was outweighed by the benefit of discussing their experiences, expressing their emotions, and having something positive come from their loss, i.e., being able to help others [15,16]. These attitudes persisted even in the face of traumatic loss, such as through suicide [17].

Thus, while there is an established body of literature examining ethical issues in bereavement research, the caveat is that it tends to be focused on adults. However, adults normally do not undergo developmental changes as children do when entering adolescence. During puberty, youth experience many changes ranging from physical to cognitive [18]. These changes can be compounded with other factors, such as the relationship between the deceased and the bereaved person [18]. For example, parental death is considered a severe if not incomparable loss for minors and can impact their development and threaten how the family functions. Alternatively, a sibling death can lead to the disenfranchised grief of the surviving minor—that is, their mourning is overlooked and unsupported, as it is not conventionally recognized such as that of parental grief [19,20]. Overall, experiencing the death of a close person is one of the most devastating events in the lives of children and adolescents with both potential short-term and long-term impacts on their mental health and social functioning, including increased risks for depression, anxiety, and substance abuse [20]. Due to the unique circumstances of children and adolescents, compared to adults, ethical issues that arise when conducting bereavement research with minors should be treated as distinct problems. Therefore, bereavement researchers should not solely rely on general ethics guidelines for human research.

The scoping review was undertaken to find what ethical issues have been identified when conducting bereavement research with minors over the last two decades (since the year 2000). By exploring this topic, this review can enhance our understanding of the ethical issues involved in this research, inform future bereavement research practices, and provide direction for further research studies on the topic.

## 2. Materials and Methods

A scoping review following the framework developed by Arksey and O’Malley [21] and adhering to the “Preferred Reporting Items for Systematic reviews and Meta-Analyses extension for Scoping Reviews” (PRISMA-ScR) guidelines (http://prisma-statement.org/Extensions/ScopingReviews, last accessed on 4 August 2022) was conducted [22]. It was decided to conduct a scoping review of peer-reviewed articles and dissertations because such reviews can be more general in terms of topic breadth and the research question posed and include various study designs [21].

### 2.1. Peer Reviewed Articles

#### 2.1.1. Eligibility Criteria

To be included in the review, studies had to: (i) be about bereavement, defined as being the death of a person the participant shared a relationship with; (ii) include minors, defined as individuals who are under the age of majority (which in most OECD countries, is set at 18 years old [23]; (iii) address ethical issues, as defined by the Australian National Statement on Ethical Conduct in Human Research 2007 (updated 2018), henceforth referred to as the “National Statement” [24]; (iv) be qualitative, quantitative, mixed methods, or a case study; (v) have been published between 2000 and 2022 inclusive; and (vi) be available as a complete text.

Studies were excluded if: (i) they were not on human bereavement; (ii) did not include minors in the sample; (iii) only mentioned general ethical concerns and procedures without providing insight into the reasoning behind the decisions made; (iv) focused on the development of new manuals or measurement instruments; (v) were review studies of non-peer-reviewed publications (e.g., books, editorials, reports, news articles, web pages); (vi) the full text was unavailable or was not in English.

#### 2.1.2. Information Sources and Search Strategy

Researcher A.E.S.P. conducted a search in Embase, Emcare, EBM Reviews, Medline, PsycINFO (all accessed via Ovid), CINAHL, Scopus, and Social Sciences Citation Index in April–May 2021, and the search was updated in July 2022. The following search string with a combination of MeSH and text words was used in Medline: (children.mp. OR Child/OR adolescents.mp. OR Adolescent/OR youth.mp. OR Minors/OR minors.mp.) AND (Bereavement/OR bereave*.mp. OR Grief/OR grief.mp. OR mourn*.mp. OR suicide loss.mp.) AND (Ethics/OR ethics.mp. OR ethics research.mp. OR Ethics, Research/OR ethics committee research.mp. OR Ethics Committees, Research/). The same search string was utilized for the other databases. No filters were used in conjunction with the search string.

Researcher A.E.S.P. also conducted a handsearch, that is, a manual search, through the online versions of Death Studies (https://www.tandfonline.com/loi/udst20, accessed on 14 August 2022) and OMEGA-Journal of Death and Dying (https://journals.sagepub.com/loi/omea, accessed on 14 August 2022) in August 2021, and updated the search in July 2022. The journals were chosen due to their unique focus on death and bereavement. It was decided that a handsearch was prudent because while the journals are included in the searched databases, only select publication years are covered, and relevant literature may have been missed during the database search due to the keywords they are tagged with. Since the review focuses on contemporary ethical issues, the volumes of Death Studies and OMEGA spanning from 2000–2022 (as of July 2022) were searched.

A handsearch of the reference lists of the peer-reviewed articles included in the review was conducted, as well as a forward citation search using Google Scholar in July 2022.

**Figure 1:**
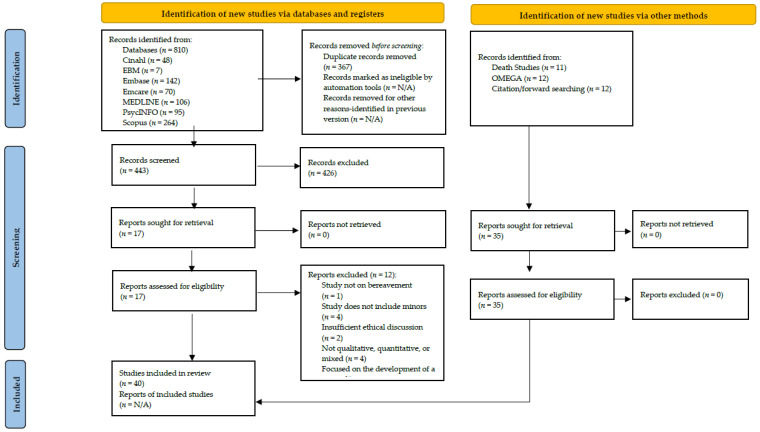
PRISMA flow diagram for peer-reviewed literature.

Following the searches, researcher A.E.S.P. deleted the doubles and screened title and abstracts of the leads. Next, researchers A.E.S.P. and K.A. screened the full texts against the eligibility criteria. Disagreements between the two researchers were resolved through an iterative process and discussion with the third researcher K.K. Figure 1 summarizes the search and selection process.

#### 2.1.3. Data Extraction

Researcher A.E.S.P. extracted the following information from the selected studies: authorship and publication characteristics (i.e., author(s), publication year), study characteristics (i.e., study purpose, design, location, setting), sample characteristics (i.e., sample size, age of the participants, sex distribution, the deceased’s relation to the participant, time since bereavement), and the ethical issues addressed in the research.

#### 2.1.4. Synthesis of Results

Data analysis consisted of a descriptive and thematic analysis. The descriptive analysis involved synthesis of information about authorship and publication characteristics, study characteristics, and sample characteristics. The thematic analysis adopted a deductive approach and utilized the framework of the National Statement [24], as outlined in Table A1, to determine the main themes.

The analysis occurred through an iterative process described by Braun and Clarke [25] and consisted of reading and rereading the articles to produce initial codes. Next, initial codes were grouped in potential themes, which we reviewed against the framework of the National Guidelines before deciding about the main themes.

Research ethics guidelines from other English-speaking countries were considered. Specifically, those from the United States (Belmont Report, 1979; the Federal Policy for the Protection of Human Subjects, aka the “Common Rule”; 2018) [26,27], Canada (Tri-Council Policy Statement, 2018) [28], the United Kingdom (UK Policy Framework for Health and Social Care Research, 2017) [29], and New Zealand (HRC Research Ethics Guidelines, 2021) [30]. However, there were no substantive differences between the guidelines in terms of what was considered to be ethically sensitive. Further, some aspects of these rejected guidelines made them less suitable for the purposes of the scoping review. The review is restricted to the years 2000–2022 inclusive; thus, it would be illogical to use the 1979 Belmont Report. The application of the Common Rule [27] is complex in that human research with federal stakeholders is subject to that of federal body’s regulations, and their head has the discretion in deciding whether the activity being carried out even falls under the Common Rule [27]. The UK Policy Framework for Health and Social Care Research [29] had portions that were of little consequence for the review, since it is organized by stakeholder responsibilities (e.g., there are sections for chief investigators, funders, organizations, etc.). As well, the legal remit of the document in regard to research involving children differs between the various countries making up the UK. The Canadian Tri-Council Policy Statement [28] was discounted, as it did not include a section focusing on research involving children, but rather mentioned it throughout the document. The New Zealand HRC Research Ethics Guidelines [30] include a section on child participants, but the discussion is less detailed than that found in Australia’s National Statement [24].

The National Statement [24] was chosen due to a number of reasons, including its use of guiding principles that are more abstract concepts (e.g., justice, see Appendix A for the principles and their definitions) to complement more concrete issues such as gaining informed consent; inclusion of sections specific to different participant groups, including children and young people; and its acknowledgement that “these ethical guidelines are not simply a set of rules. Their application should not be mechanical. It always requires, from each individual, deliberation on the values and principles, exercise of judgment, and an appreciation of context” [24].

### 2.2. Dissertations

#### 2.2.1. Eligibility Criteria, Information Sources and Search Strategy

A preliminary search through relevant handbooks/chapters revealed that they tended to offer little beyond a general overview of ethics. As such, it was decided to limit the search to dissertations. We applied the same inclusion and exclusion criteria as for the peer-reviewed articles. A search was conducted using ProQuest Dissertations and Theses Global, as it was identified as hosting the most extensive collection of full-text theses worldwide (https://www.proquest.com/index, accessed on 14 August 2022). The search was conducted in September 2021 and updated in July 2022, with the following search string imputed into the “advanced search” function: ab(bereave*) AND ab(children OR adolescent* OR minors OR youth) AND ethic*. No filters were used in conjunction with the search string.

**Figure 2:**
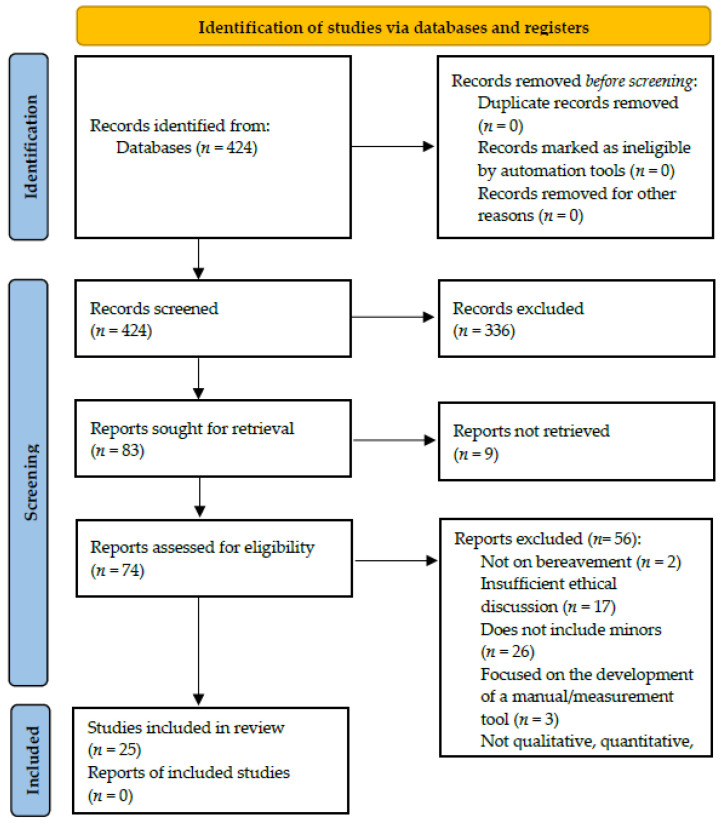
PRISMA flow diagram for dissertations.

Researcher A.E.S.P. conducted the searches and screened the leads based on title and abstract. Researchers A.E.S.P. and K.A. screened the full text of the selected leads. Undetermined cases were resolved through discussion with the third researcher K.K. Figure 2 summarizes the search and selection process.

#### 2.2.2. Data Extraction

Researcher A.E.S.P. extracted the following information from the selected dissertations: authorship and submission characteristics (e.g., author(s), submission year, country of origin), study characteristics (e.g., study purpose, design, location, setting), sample characteristics (e.g., sample size, age of the participants, sex distribution, the deceased’s relation to the participant, time since bereavement), and the ethical issues addressed in the research.

#### 2.2.3. Synthesis of Results

The method of data analysis for the dissertations was the same as that used for peer-reviewed literature.

## 3. Results

### 3.1. Peer Reviewed Articles

#### 3.1.1. Study Characteristics

A total of 40 articles were included in the review (Table A2). Four studies were from Australia [14,31,32,33], eleven from the US [34,35,36,37,38,39,40,41,42,43,44], seven from Sweden [45,46,47,48,49,50,51], four from Norway [52,53,54,55], four from Denmark [56,57,58,59] four from the UK [60,61,62,63], and one from Canada [64]. There were five studies of non-western origins: two from South Africa [65,66] one from China [67], one from Iran [68], and one from the Philippines [69]. Four studies were published in the 2000s [41,42,52,53], 22 in the 2010s [31,35,36,37,38,39,40,43,47,48,49,54,55,57,58,60,61,62,63,64,65,66], and 14 from the 2020s [14,32,33,34,44,45,46,50,51,56,59,67,68,69]. Most studies were qualitative in design; there were also five quantitative [42,43,46,50,67], and five mixed-methods studies [14,45,51,52,62].

Overall, the age ranges of the young participants varied widely, with the youngest participants aged 4 years [64], and there were 10 studies that included participants over the age of 18 but categorized them in the child/youth groups [14,31,32,33,37,44,46,52,56,62]. Parents and other adult figures were commonly included in the studies as participants [14,31,33,37,39,41,42,44,45,49,50,51,52,53,55,59,60,63]. 

#### 3.1.2. Ethical Values and Principles

Most studies identified multiple ethical issues, with the most common concerns regarding consent [31,32,33,37,40,46,56,57,63,64,67,68,69], potential risks and mitigation strategies [14,34,35,36,37,45,53,56,58,59,60,65,66,68,69], and privacy and confidentiality [34,38,41,45,46,52,56,59,60,63,65,68]. Table A2 in Appendix B details the study characteristics and the ethical issues found in the peer-reviewed studies.

### 3.2. Dissertations

#### 3.2.1. Study Characteristics

A total of 25 records were included in the review (Table A3). Eighteen studies were from the US [70,71,72,73,74,75,76,77,78,79,80,81,82,83,84,85,86,87], one from the UK [88], two from Canada [89,90], one from Sweden [91] and three from South Africa [92,93,94]. Thirteen studies were conducted in the 2000s [70,73,75,76,77,78,81,82,84,87,89,90] and fifteen in the 2010s [71,72,74,79,80,83,85,86,88,91,92,93,94]. Most studies were qualitative studies. Five studies were quantitative [71,74,75,77,81], and five other studies used mixed methods [76,78,84,85,91].

The age ranges of the participants varied, with the youngest participants aged 3 years [83], and five studies including participants over the age of 18 in the youth groups [82,88,91]. Some studies included adult respondents as well [70,73,78,79,80,81,84,85,87,90,91,93].

#### 3.2.2. Ethical Values and Principles

Most studies identified multiple ethical issues. The most common principles were privacy and confidentiality [71,72,73,74,76,78,79,82,83,84,86,87,88,89,90,91,92], informed consent [70,72,73,78,80,83,84,85,86,88,89,91,92,93], and potential risks and mitigation strategies [72,73,75,76,77,80,84,86,88,89,91,92,93,94]. Table A3 in Appendix B presents the study characteristics and the ethical values and principles identified in the dissertations.

### 3.3. Overall Results Regarding the Handling of Ethical Issues

The ethical issues found in the 40 peer-reviewed articles and 25 dissertations can be categorized into overarching themes: privacy and confidentiality, informed consent, and potential risks and associated mitigation strategies.

#### 3.3.1. Privacy and Confidentiality

Some researchers used intermediaries, such as support services or school counsellors, to recruit participants [46,67,68,71,73,74,78,86,88]. There were instances when permission from third parties such as government bodies was needed in order to access data [52]. In other instances, the law deemed information public [43,44]. Disclosure and maintaining the confidentiality of data shared by minors unless there were extenuating circumstances such as report of abuse/neglect was an important issue [45,59,63,94]. Keeping confidentiality was not always the default however, and permission from the minor’s guardian to do so had to be sought [73]. Family studies also posed issues of confidentiality between family members if group interviews were held [41,73]. Researchers commonly assigned pseudonyms or identification numbers, and withheld personal identifiers [38,47,48,49,59,69,71,72,76,78,79,80,83,84,86,87,90,91,92,94,95]. Some participants forewent the use of pseudonyms however [86,92].

A less common was the issue of the ownership of the generated information/artefacts [65,94]. For example, in cases of interventions involving memory work with orphans, the information being gathered is private, as it is family history. As such, the ownership of the information ultimately resides with the participants, who have control over the “product”, and its presentation and access [65]. Regarding more tangible products such as artwork produced by the participants, it could be seen as the researcher temporarily borrowing them for their studies. In which case, there are issues related to the storage and preservation of the artefacts to ensure that they are not altered/damaged, and how analysis is carried out (e.g., unable to mark the artefacts), which in turn can lengthen the analysis process [94].

#### 3.3.2. Informed Consent

The issue of obtaining informed consent focuses on the fact that the participants are minors, and there are both legal and developmental factors to be considered. There were various approaches in how researchers gained consent for minors. They obtained consent from the participant’s guardian [54,87]; sought both guardian consent and participant assent [35,37,38,40,42,43,44,47,52,53,55,56,57,64,65,72,74,76,79,83,84,85,86,90,91,92,95]; or they judged on a case-by-case basis if the participant was cognitively mature enough to provide consent, given that they first reach a certain age threshold, such as age 16 [31,32,33], and between 7 and 17 [64].

Adherence to the law was also a factor in obtaining consent from minors [45,46,51]. There are times consent is assumed, since the data are judged to be in the public sphere, such as chat threads on open forums [46,47,48]. The power dynamics between minors and adults (i.e., researchers, parents, etc.) and how it could influence their decision to participate was of concern [72,80].

#### 3.3.3. Potential Risks and Mitigation Strategies

Researchers were focused on mitigating risks to participants rather than the potential benefits to justify participation. Participants would be screened, for example, based on factors such as time since bereavement [54,58,88,95], assessment by others such as the partner organization or professionals who referred the participant [87,89], self-assessment [34,80], or whether the bereavement was due to traumatic reasons [59]. Collaboration between researchers and other parties such as interventionists and healthcare professionals allowed for better monitoring of participants’ well-being [45,53,60,62]. Participants’ mental/emotional states were assessed after taking part in the study, and in some studies, there would be further follow-up [35,68,92]. Participants could also approach researchers, or they were offered references to professional support services should they experience distress during or after the research [36,37,62,66,69,72,73,75,78,86,92].

There were few studies that also factored in the well-being of the researchers and other involved third parties such as program facilitators. Vaswani [63] suggested that since bereavement researchers employ skills similar to therapists, they also should adopt methods that therapists employ to maintain their well-being. Researchers relied on support from others such as peers, one’s academic supervisor, or agencies [86,88]. In regard to other involved parties such as program facilitators, it was the responsibility of the individuals themselves as well as their organizations to ensure their well-being through means such as training; the researchers had no part in this aspect [65].

## 4. Discussion

This scoping review was conducted to answer the question of what ethical issues have been identified when conducting bereavement research with minors, using the Australian National Statement [24] as a background. An extensive search through peer-reviewed literature and dissertations found 68 relevant studies (40 peer-reviewed, 25 dissertations). The studies differed in terms of purpose, participant demographics, time since bereavement, and research setting. There were some commonalities of note between the studies, however, specifically in publication origin (location). This review focused on studies published over the last two decades (2000–2022). It appeared that discussion of ethical issues has become more commonplace in recent years, which may indicate an increasing awareness for ethical issues in this field.

Modern research ethics was established, as a response to the human experimentation conducted during World War II, in the form of the Nuremberg Code (1946). The Nuremberg Code served as a foundation for the Declaration of Helsinki (1964). Unfortunately, these guidelines were not strictly regulated, nor was there any incentive to abide by them. However, to use the US as an example, knowledge of research ethics violations on home soil spurred change due to public outcry. Details of the Tuskegee Syphilis Study would become public in the 1970s. This would lead to the establishment of the National Commission for the Protection of Human Subjects of Biomedical and Behavioural Research, whose recommendations would take the form of the Belmont Report. The Belmont Report was used for federal regulations and was continually revised until 2001 [95]. The Belmont Report informed the Federal Policy for the Protection of Human Subjects, colloquially known as the “Common Rule”, [27], currently in use in the US. Research funded by any of the signatory agencies must abide by it. Even if funding is not involved, some states may have laws that incorporate the Common Rule. Most educational institutions in the US utilize the Common Rule as a matter of institutional policy. Compliance can be reinforced via employment contracts that refer to these policies, making it a matter of employment law [96]. The US is not the only country to tie funding and legislation with adherence to research ethics guidelines. The previously mentioned guidelines of Canada [28], Australia [24] New Zealand [30], and the UK [29] do so as well to varying degrees.

Most studies and dissertations originated from western countries. There may be various factors for this seeming disinterest from non-western countries in ethical issues in bereavement research. One factor may be attributed to developing countries’ lack of capacity for ethics review committees [97]. Another factor can be differences in cultural values and beliefs. These differences can lead to unique interpretations of research ethics that were founded on Western principles [98,99]. There may also be a difference in research priorities.

In general, the ethical issues are addressed straightforwardly in published articles, with authors listing the steps they have taken in the design/process to make the research comply with ethical guidelines. It was uncommon for any in-depth reporting on concerns or analysis on why certain measures were taken. In the dissertations, on average, the discussion of ethical issues was more detailed than in peer-reviewed articles, seeing as most of the dissertations had sections solely devoted to ethical considerations. The difference may largely be a matter of practicality. With journals that have word/page limits in place, authors may prioritize reporting data analysis and results rather than discussing ethical quandaries if they are not the focus of the research.

Comparing the results of this scoping review to the ethical issues discussed in the literature regarding bereaved adults [1,3], it is evident that privacy and confidentiality, informed consent, and risks and benefits are common concerns regardless of the age of the participants. Looking at research involving minors from other fields (e.g., social research) for comparison, informed consent and confidentiality are topics of particular interest as well [100,101]. The specifics and importance of these concerns may differ between minor and adult participants. These differences can be attributed to how adults perceive minors, which in turn affects the research. Adult perception of minors is influenced by childhood being marginalized such that adult–child power dynamics are unequal; pre-conceived notions and attitudes towards children; and differences that may be attributed to developmental factors [102]. There are also larger forces at work beyond the attitudes of individual adults. Ironically enough, ethical issues can arise from the use of research ethics guidelines. This can be attributed to the fact that, commonly, they were not based on a child-centric perspective. There can also be a mismatch because the framework for the guideline might not be suitable for bereavement research [103]. Overall, the ethical issues of bereavement research with minors are treated as a common hurdle for researchers to resolve. However, future studies should also consider these larger, institutional factors to better inform research practice.

This review demonstrates there are many considerations when conducting ethical bereavement research involving minors. However, the extent to which the issues identified are specific to bereavement research, or are merely common concerns found in any research involving minors deemed to be sensitive, is debatable. In a Delphi study on identifying ethical issues in mental health research involving adolescents [104], some of the most common issues were related to consent and confidentiality, which is in line with what was found in this review. However, the issues discussed in that Delphi study [104] are more nuanced. For example, in certain bereavement studies, it was permissible for participants who were 16 years of age or older to give consent without the additional consent of their guardian necessary, provided the researchers deemed them competent to do so [31,32]. However, this was the only circumstance found in which guardian consent was circumvented.

This is in contrast with the Delphi study [104], which provides further extenuating circumstances in which adult consent may be waived, including: if there may be significant benefits for the minor in participating; there is minimal risk associated with the study and other details of the study (e.g., the research topic is of sensitive nature, such as that of familial relationships); the status of the guardian and/or the minor (e.g., the adult themselves are unfit to give informed consent/the minor is evaluated as being cognitively capable); and extenuating circumstances (e.g., participation is a matter of life and death) [104]. This difference is relevant, as it raises the question why such nuances were not found in the bereavement studies, considering that many involved the experiences of the bereaved youth, and ties in with their mental health (e.g., [31,43,50,74]).

In general, the overall sense is that the underlying issue with bereavement research involving minors involves a struggle of safeguarding participant well-being while not being paternalistic. Children and adolescents are viewed as those in need of protection, and as such, extra measures are taken to ensure that they are not put at risk. Further, death, while not a taboo in Western countries, is still deemed a sensitive topic. Death is ever present in the media. However, human research ethics committees and gatekeepers alike persist in being reluctant for the subject to be broached with minors, even in the face of evidence of the potential benefits. The overly cautious approach taken may, ironically enough, send the signal that death is something to hide when the intent of research is the opposite [90].

### Limitations

There are limitations to the review. In terms of the evidence found, the perspectives presented in the literature are relatively homogenous in nature, since the majority of the studies originated from western countries. In addition, most studies were qualitative in design, which raises the question of why quantitative and mixed methods studies do not report ethical issues as frequently. Finally, certain information (e.g., sample size, age, sex distribution, and time since bereavement) was not regularly reported in the peer-reviewed literature, which makes it harder to spot data trends.

The scoping review did not include an appraisal of the quality of the included studies due to the focus of the review question being exploratory in nature. As such, the methodological quality and risk of bias in the included studies are unknown [105]. There is also room for debate regarding how the ethical issues are categorized, as only the guiding principles of the National Statement on Ethical Conduct in Human Research 2007 (updated 2018) [24] and the issues they outline were utilized for the scoping review. Future reviews may also examine how the ethical issues reported in this review are similar or different to those reported in research with minors in other fields.

## 5. Conclusions

This scoping review identified the common ethical issues in bereavement research involving minors. These issues tend to fall under the principles of informed consent, risks and potential benefits, and protection of privacy and confidentiality. The review also identified the methods used by researchers in addressing these issues. Going forward, the scoping review could be used by bereavement researchers when designing their studies and to address ethical issues as they arise. The review can also be used to support the decisions researchers make to a human research ethics board, thus contributing to the literature on this subfield.

## Data Availability

Data from this study are Available online the researchers upon reasonable request.

## Appendix A

**Table A1:** Summary of the principles and themes of the Australian National Statement on Ethical Conduct in Human Research 2007 (Updated 2018) [23].

**Principle**	**Scope**
Research merit and integrity	For the research to have merit it must: Have potential benefit Designed using methods fitting for the proposal Based on current literature and previous studies (not excluding novel research) Not compromise the respect due to participants Conducted/supervised by qualified individuals Conducted using appropriate facilities and resources Integrity: Depends on the motivation and conduct of the researchers
Justice	The research design must be fair, and results accurately described The recruitment process is fair, with no undue burden on particular groups of participants Benefits must be distributed fairly Not exploit participants Research outcomes are accessible in a timely and clear manner to participants
Beneficence	The benefits of conducting the study outweigh possible repercussions to the participants, who must be informed of said potential benefits and risks The researchers have a duty towards the participants to design the study in such a way to minimize risks and clarify to them the potential risks and benefits. The researchers are responsible for the welfare of participants during the study If there are no possible benefits, the risks must be lower than if there was anything to be gained In the event the potential risks are no longer justifiable, the research must be suspended for review
Respect	Recognizing the inherent value participants hold as humans, and as such holding their welfare, beliefs, perceptions, and culture and customs in regard Respecting the privacy, confidentiality, and cultural sensitivities of participants (and their communities); any specific agreements made with the participants/community should be honoured Acknowledge and allow for participants to exercise their ability to make decisions, which is within their capacity as human beings If participants are unable to make their own decisions/have diminished capacity, empower them and provide protection
**Themes**	**Scope**
Risk and benefit	Assessment of risk Identifying any risks Gauging possibility and severity Assessing the extent they can be minimized Determining whether they are justified by the potential benefits Determining how they can be managed Potential harms include physical, psychological, devaluation, social, economic, and legal Harm is the most serious outcome, followed by discomfort then inconvenience “Low risk research” = only foreseeable risk is discomfort “Negligible risk research” = no foreseeable risk of harm or discomfort
Consent	Consent should be voluntary and based on sufficient information and understanding of the research and the implications of participating Information must be presented in a suitable manner for each participant The aim of conveying information and gaining consent is mutual understanding between researchers and participants. Participants should be able to ask questions and discuss the opportunity with others as they wish There are different ways consent can be expressed, and they depend on factors surrounding both the research and the participant’s circumstances Consent should be renegotiated especially when projects are complex, long running, or participants are deemed to be vulnerable There should be no coercion and pressure Participants should be made aware of: Alternatives to participating How the research will be monitored The services available if the research negatively impacts them and they require additional assistance Contact details of the researchers as well as the person charged to register complaints How privacy and confidentiality will be protected Their right to withdraw from the study and whether their data can be withdrawn Financial information and/or declaration of interests for all parties involved Any payments The likelihood and form of dissemination of the research results The expected benefits to the wider community Reimbursement Generally appropriate to reimburse the costs of taking part in research, including those pertaining to transport and accommodations. Sometimes participants may also be paid for time involved. However, payment/other inducements that are disproportionate to the research involved and encourages risk to be taken is unethical Decisions about payment/reimbursement should take into account the practices of the community the research is being conducted in When a potential participant cannot give consent, a third party with the legal authority to make decisions on behalf of the individual should be approached with the relevant information instead. Their decision must be in the best interest of the potential participant.

## Appendix B

**Table A2:** Summary of peer-reviewed literature.

Author, Publication Year, Study Origin	Study Purpose	Sample Size	Age	Male/Female	Deceased’s Relation to Participant	Time since Bereavement	Setting	Study Design	Ethical Issues
Andriessen et al., 2018 Australia [31]	Examining the experiences of bereaved adolescents seeking help	*N* = 39	13–27	30 females 9 males	Family member, friend	6 months–10 years	Phone	Qualitative	The consent procedure was adapted per the guidelines in the National Statement on Ethical Conduct in Human Research. For participants aged 12–15, consent from their guardian was sought. For those aged 16–17, it was left to the researcher’s discretion, after the initial meeting, whether the participant was mature enough to give informed consent or their guardian’s consent was needed. In unclear cases, a second opinion was sought from a co-investigator. Reimbursed with a gift voucher.
Andriessen et al., 2020 Australia [32]	Examining the experiences of adolescents bereaved by a traumatic death	*N* = 38 (20 adolescents, 18 mothers)	14–26 (adolescents) 43–60 (mothers)	16 females, 4 males (adolescents) 18 females (mothers)	Family member, friend	1–10 years	Phone (for individual interviews) N/A (for in-person group interviews)	Qualitative	Due to the age range, the issue of obtaining consent was of concern. To that end, the researchers adapted their consent procedure per the guidelines laid out in the National Statement on Ethical Conduct in Human Research 2007 (updated 2018). For participants aged 12–15, consent from both participant and their guardian was sought. For those aged 16–17, it was left to the researcher’s discretion, after the initial meeting, whether a guardian’s consent was necessary. Reimbursed with a gift voucher.
Andriessen et al., 2021 Australia [33]	Examining the kinds of support suicide-bereaved adolescents find helpful	*N* = 18	14–23	14 females 4 males	Family member, friend	1–9 years	Phone (individual interviews) N/A (in-person group interviews)	Qualitative	Sought parental consent for participants under 16 years old. For those who were 16–17, it was decided on an individual basis by the lead researcher, after the initial meeting, if parental consent was necessary. Reimbursed with a gift voucher.
Andriessen et al., 2022 Australia [14]	Examining the short-term impact of participating in research focusing on traumatic death	*N* = 72 (20 adolescents, 18 parents, 34 clinicians)	14–26 (adolescents) 43–60 (parents) 26–71 (clinicians)	16 females, 4 males (adolescents) 18 females (parents) 28 females, 6 males (clinicians)	Family members and friends (adolescents) Family members (parents)	1–10 years	N/A (provided a hard copy of survey to be mailed back/emailed the survey to be returned)	Mixed method	Results indicate adolescents are likely to experience the greatest distress when participating in research interviews. Researchers note that this reinforces the need for informing potential participants on possible risks that come with taking part in the research; and for having appropriate measures such as distress protocols in place. Mentions how ethical issues may occur if the boundaries between research and therapy are not observed. Important for interviewers to be qualified and able to handle participant’s emotions. Must also be able to make judgment calls on whether an interview can proceed or if the participant should be referred to third party support systems.
Asgari, Naghavi, and Abedi 2022 Iran [68]	Exploring the experiences of Iranian adolescents who lost their parents due to the COVID-19 pandemic	*N* = 15	14–18	8 females 7 males	Parent(s)	N/A	Online (via video conferencing)	Qualitative	Schools/organizations only gave participant information after receiving their consent. However, three participants withdrew their consent due to mental health issues and were instead provided referrals. After each interview, the participant’s emotional state was assessed. Further follow-up was performed via text. Participants were given pseudonyms.
Barney and Yoshimura 2021 US [34]	Exploring themes related to the process of sorting the belongings of a deceased relative	*N* = 32	16–79	23 females 9 males	Relative	1–10.5 years	Private locations (unspecified); videoconferencing	Qualitative	Minimized discomfort/risk by excluding individuals who became bereaved less than a year ago and felt they had grief-related trauma that could potentially be triggered during participation. Identifying information was removed, and participants were assigned pseudonyms.
Coullie 2018 South Africa [65]	Examination of the memory box project run by the Sinomlando Centre for Oral History and Memory Work in South Africa	N/A	N/A	N/A	Primary caregiver	N/A	Participants’ homes, Sinomlando Centre	Qualitative	Obtained during the group discussions. Memory workers must be able to communicate in the home language of the program participants, and training workshops are geared towards fostering an appreciation of the circumstances the families and their communities face. Respect for the participant’s culture must be shown (e.g., skirting around the topic of AIDS/HIV when necessary, since it is a taboo subject) but at the same time bent in that by participating in the memory box project, children are afforded privileges not normally given to them (social norms are that children are discouraged from asking questions and are not informed about matters that have to do with adults). Some of the methods used in the program are from fields such as psychology, and the question of whether or not this reinforces “colonial knowledge paradigms” comes into play. Intrusiveness of the methods used is balanced by respect being shown towards the participant’s cultures and traditions and facilitators bolstering the dignity of the participants. The primary beneficiaries of the memory boxes are the participating families. Privacy is upheld since the memory boxes are not created with the intent to extract research data. As well, facilitators are instructed to hold discussions with the families in regard to confidentiality and how the information recorded belongs to them. Minimise distress by practicing sensitivity in deciding upon who to involve in the project and when. Facilitators are trained in how to handle sensitive memories and the emotions that may arise during participant’s recollections. Part of this is by ensuring participants are always aware that they can withdraw from the intervention at any time, and referring the families to a specialist when possible. Facilitators must also ensure that there are no false expectations raised in regard to what is guaranteed by participating. The facilitators have an ethical duty towards themselves as well to ensure their own well-being, and this is addressed through their training workshops which includes self-reflection and being made aware of available resources. Facilitators also meet to discuss the program and are able to voice concerns and gain advice in this manner. The ethics of memory and the role it plays in identity formation.
Dyregrov and Dyregrov 2005 Norway [52]	Exploring the state of siblings bereaved by suicide	*N* = 198 (70 siblings, 128 parents)	15–20 (sibling subsample 1) 21–43 (sibling subsample 2) 36–73 (parents)	5 females, 6 males (sibling subsample 1) 39 females, 20 males (sibling subsample 2) 77 females, 51 males (parents)	Child/sibling	Late 1990s	Participants’ homes	Mixed method	Had to seek permission from the Norwegian Ministry of Law and Justice in order to access the national policy register. Gained additional approval from the attorney general, the council for professional secrecy and research, and the data inspectorate of Norway for the study. Parents and siblings were informed about the project, and with understanding about anonymity and provided gave written consent. The attorney general did not give permission for inquiries into those who did not respond back beyond the information already available in the register, citing confidentiality.
Eklund et al., 2021 Sweden [45]	Exploring the perception of families who participated in in an intervention focused on the imminent death of their family member during the illness and after loss	*N* = 26 (8 parents, 18 children)	8–24 (children) 39–54 (parents)	7 females, 9 males (children) 6 females, 2 males (parents)	Partner/parent	1–10 months	Participants’ homes	Mixed method	Healthcare workers determined eligible families who could be potential participants. Researchers met with prospective families and provided information (both written and verbally) and allowed them to ask questions. The information was adapted to suit the comprehensive levels of children. Participants made aware they could quit at any time, and that they could also attend the intervention and study even if the parent died. Participants were assured of confidentiality—identifying information would be altered/redacted and other family members would not be informed of what was shared (aside from information about potential harm the child has suffered, in which case their parents and other relevant individuals would be notified). Guardian consent was needed for participants under 15 y/o, while those who were older gave consent per Swedish law. Researchers had in place plans if information concerning potential harm to a participant was revealed. As well, the researchers, interventionists, and healthcare professionals maintained cooperation so that they knew who was participating in the research and could provide support to those who reported distress.
Feng and Lan 2020 China [67]	Exploring behaviour changes of familial bereaved adolescents	*N* = 763	13–18	60.3% females	Grandparents or siblings	M = 4.57 years	Participants’ classrooms	Quantitative	Obtained permission from school principals. Asked head teachers to send out information through messaging app to inform parents about the research and participants’ rights. Gained consent through an opt-out method, with parents having to contact the teacher if they did not want their child to participate in the research. Confidentiality and anonymity said to be guaranteed (but no details given). Adolescents asked if they wished to participate. If they declined, then they were given other work to do by their teachers.
Fjermestad et al., 2008 Norway [53]	Exploring how orphans and their caregivers cope, with support from a community-based program	*N* = 16 (8 orphans, 8 caregivers)	12–16 (orphans) Youngest caregiver reported to be 18	4 females, 4 males (youth) 8 females (caregivers)	Parent(s)	N/A	Community organization office, participants’ homes, local church	Qualitative	An information meeting was held for the children to explain the study and to see who was interested. Caregivers signed consent forms for themselves and their charge. The children gave consent as well. Meeting location varied, according to the informant’s preference. Anonymity and confidentiality were assured (no further details given). Some of the topics could elicit negative emotions and were culturally sensitive. Participants were informed that they could refrain from answering questions and/or end the interview if they felt the need to. There was also follow-up support in place, and the researcher monitored the participant’s mental state during the interviews.
Hansen et al., 2016 US [35]	Exploring bereaved adolescents’ continued bonds with their deceased parent	*N* = 15 (9 adolescents)	12–18	N/A	Partner/parent	N/A	Participants’ homes	Qualitative	Gained consent from parents for both themselves and their children, and children provided assent. Participants who exhibited distress were given the option to end the interview. After the interview, participants were assessed for distress. Participants received an honorarium after providing consent.
Heffel et al., 2015 US [36]	Examining the role of social media after a suicide cluster	*N* = 10	Avg. age 16.7 years	4 males 6 females	School peers	1 year	School	Qualitative	No randomized selection of participants, instead the researchers consulted with one of the high school counsellors to identify potential participants. As well, all students in the district were offered services at a local mental health clinic. Participants in the larger study had the opportunity to enter a raffle for prizes.
Holm et al., 2022 Sweden [46]	Exploring avenues of support and their importance for bereaved families with children	*N* = 65 (42 parents and 23 adolescents who completed the questionnaires)	34–60 (parents) 12–19 (adolescents) 4–11 (children reported by proxy)	24 females, 18 males (parents) 15 female, 8 male (adolescents) 11 females, 16 males (children reported by proxy)	Partner/parent	~1–3 years	Online or other (for those who completed paper versions of the questionnaires)	Quantitative	Explicitly acknowledged the fact that “because children and adolescents were involved, it was considered especially important to balance the possible harm of the project against the scientific knowledge gained (p. 997).” The research group did not have access to the eligible families’ contact information; rather, Statistics Sweden sent out information letters to them. Researchers could only contact potential participants if they signed up. Parents provided consent for themselves and their children. Participants aged 15 years and older could consent and participate, regardless of their parent’s involvement. All minors had to provide assent, per Swedish law.
Holmgren 2021 Denmark [56]	Study of bereaved families and their experiences following the death of their intimate partner/mother	*N* = 11 (4 men, 7 children)	men: N/A Children: 7–19	4 men 3 girls, 4 boys	Intimate partner/mother	1–2 years	Family homes	Qualitative	For participants under the age of 18 the minors assented while written consent was also obtained from fathers. The researcher informed the children that they were allowed not to answer questions and to withdraw from the study at any time. In most cases, the children and fathers were interviewed separately so that they could freely express themselves (one child did want their father present during their interview). The interviews were conducted in the participants’ homes with the aim of making the interview less stressful and formal. In addition, the fathers were generally interviewed first so that the researcher could get a better understanding of the family background and be informed of any particular concerns regarding the children. The researcher acknowledged that there is a power imbalance between themselves, an adult, and the children. This was addressed by stressing the fact that the child was the expert, since there was no “correct” answer, and the focus was on their experiences, thus empowering them. As well, interviews were conducted with the child’s age and cognitive abilities in mind.
Johnson 2010 US [37]	Examining the experiences of bereaved female African American adolescents who lost friends through homicide	*N* = 20	16–19	20 females	Friend	1 month-6 years	N/A	Qualitative	The researcher explicitly acknowledged “research on a sensitive subject with African-American teen girls, some under 18 years old, required attention to specific ethical considerations.” Participant assent was collected, along with parental consent for participants under the age of 18 and those referred through public schools (ignoring age). Participants over the age of 18 and not recruited through the schools gave written consent. Participants were offered access to counselling. Given monetary compensation and a certificate of appreciation for participating.
Jonas-Simpson et al., 2015 Canada [64]	Exploring sibling-bereaved children’s experiences regarding familial and school life	*N* = 14 (5 parents, 9 children)	4–17 (children)	5 males, 4 females (children) 2 males, 3 females (parents)	Child/sibling	2.5–12 years	Participants’ homes	Qualitative	All minors provided verbal assent, and 7–17 year olds who were deemed mentally capable of giving informed consent. Parents also provided consent for themselves and their children. Utilized volunteers at a bereavement organization to spread flyers of the study to potential participants. Administration was also asked to recommend families. Researcher talked with parents to answer questions.
Lytje 2018a Denmark [57]	Examining the experiences of parentally bereaved minors as they return to school	*N* = 39	9–17	N/A	Parent	N/A	N/A	Qualitative	Dilemma between giving children a voice and following Danish law which requires parental consent to be obtained if the participant is under the age of 18. In the end, the researcher gave a form to guardians stating they would leave the decision of participating up to the child. Afterwards, an introductory video regarding the research was shown to the minors, who were asked after viewing if they wished to participate. If they did, then the project was discussed with them one more time before they signed a form.
Lytje 2018b Denmark [58]	Examining the experiences of parentally bereaved minors as they return to school	*N* = 39	9–17	N/A	Parent	N/A	N/A	Qualitative	Excluded children who were bereaved due to traumatic deaths, since their needs may differ from those who were bereaved through less traumatic means, and it would be unethical to have them participate without additional support available. As well, the selection criteria in regard to time since bereavement was included in part because it was thought the participants would be suffering from less acute grief.
Lytje, Dyregrov, and Holiday 2022 Denmark [59]	Exploring the experiences of parentally bereaved young children	*N* = 12 (children) (Parents also interviewed)	5–8	Not explicitly stated, only provides pseudonyms	Parent	1–4 years	Participants’ homes	Qualitative	Recruited using support networks affiliated with the Cancer Society and through social media. Participants who experienced more complicated/violent deaths were excluded on the basis that the interviews “were deemed ethically problematic, as they might reopen traumatic experiences and require more substantial post-interview support than was available (p. 3).” Used an ethical framework specific to counselling and psychotherapy for the study—built rapport with the child, made the parent and child aware participation is voluntary and that they can withdraw at any time. Explanations were given in a manner appropriate for the child’s understanding. Also followed the European General Data Protection Regulation. Underwent an internal ethics approval process through the primary research organization, even though Denmark only requires this if biological material is involved. In the event the participant raised a concern during the interview, they were offered help in addressing said worry with their parent. If the child declined, then additional support would be offered to the family, while maintaining the child’s privacy. Pseudonyms assigned to participants and location data omitted. For ethical reasons, data from parent interview separated from the children’s and covered in a different article.
Mcclatchey and Wimmer 2012 US [38]	Identifying aspects of a bereavement camp that assisted in the healing process for participants	*N* = 32 (13 adults, 19 children)	8–18 (children)	Not stated, only provides pseudonyms (children) 12 females, 1 male (adults)	Partner/parent(s)/grandparents (primary caregivers)	N/A	Participants’ homes	Qualitative	PI was the founder and director of the bereavement camp. Adults signed consent forms and children gave assent. Participants received a gift certificate upon completion of the interview. They were previously unaware of the reward though. Pseudonyms were utilized in the study.
Metel and Barnes 2011 UK [60]	Examining the usefulness of a peer-group support bereavement program	*N* = 42 (originally 25 children, 17 parents. However, 2 children, both age 9, were not interviewed upon parent’s request)	8–17 (children)	Originally 15 females and 10 males (children) 14 females, 3 males (parents)	Partner/parent, sibling	M = 2.7 years	Participants’ homes	Qualitative	Parents gave consent for a home visit and confirmed with their children their interest in participating. During the visit, both parents and children gave written consent. Assured of confidentiality through an information sheet and prior to written consent. For confidentiality reasons, potential participants were approached by the programme. If they showed interest, the researcher’s contact information was passed on to them. All information, barring date of service used, was unavailable in regard to the families who chose not to participate in the research. The community-based charity (Jigsaw4U) arranged for clinical support if any of the participants became distressed.
Ofreneo et al., 2020 Philippines [69]	Examining orphans’ traumatic memories and how they reclaim agency Identification of social structures that marginalize orphans	63 in total participated in the psychosocial intervention 5 were excluded from the memory work due to age	11–18	29 boys 29 girls	Parents	N/A	Communities with faith-based organizations present	Qualitative	Researchers and memory workers had to undergo ethics training before the project commenced. The research process and ethics protocol was explained to participants and their guardians. Afterwards, written assent and consent was collected from the participant and their guardian, respectively. Psychologists and social workers were available during the research in case any participants showed distress. Transportation and meals were arranged for the study duration, and a token of gratitude was given to the participants. Participants were given pseudonyms, and references to their communities were withheld. Did not go through an institutional research ethics review; justification given is that due to the current situation in the Philippines, where human rights are violated, the authors were spurred by a sense of urgency.
Pangborn 2019 US [39]	Exploring how taking part in family story telling helps adolescents in acknowledging and managing their grief	N/A	N/A	N/A	relative	N/A	Bereavement camp	Qualitative	Mentioned procedural and situational ethics. Acknowledged that due to the experiences being shared, emotional attachment to the participants would not be a hinderance in the case of this study. Also recognized influence they (the researcher) might have on the participants, even if their time together is limited.
Parsons, Botha, and Spies 2019 South Africa [66]	Examining the experiences of maternally bereaved children	*N* = 22	10–12	N/A	mother	More than 12 months	N/A	Qualitative	Explicitly acknowledged that “rigorous efforts were made to meet the most stringent requirements of ethical research, including creating and maintaining the environment of respect (p. 5)” Guardian and child consent collected; told that they could withdraw at any time. Participants were able to request access to support services if they found themselves to be distressed due to the topic of the study. Employed models and strategies dealing with trustworthiness.
Rolbiecki, Washington, and Bitsicas 2019 US [40]	Exploring the use of digital storytelling as a bereavement intervention	*N* = 14 (2 adolescents)	24–64 (adult) 12–15 (youth)	11 females, 3 males (adults)	Child, parent, partner, sibling, other	N/A	Workshop location	Qualitative	Partnered with bereavement counsellors, a local palliative care program, churches, and a hospice agency to find potential participants. Research was deemed to be minimal risk, so consent was gathered verbally; also obtained reconsent throughout the study. For minors guardian consent was needed and child assent was also collected. Participants received compensation at two different points of the study (once after the digital storytelling workshop and another upon completion of the follow-up measures).
Saldinger, Cain, and Porterfield 2004 US [41]	Exploring attachment to a dying parent and continuing bond after their death	*N* = 58 (children)	6–16 (children) 33.7–55.3 (parents)	52% female, 48% male (children)	Partner/parent	8–36 months before study	Participants’ homes	Qualitative	Initial contact through funeral homes, a hospice organization, and newspaper advertising. Family members were individually interviewed in order to ensure confidentiality.
Silvén Hagström 2017 Sweden [47]	Examination of online collective story telling by suicide-bereaved youth	N/A	N/A	N/A	Parents	N/A	Online	Qualitative	Ethical issues surrounding netnography include whether the material is classified as public, private, or a mix of both-in this case public since the chat could be read and replied to by anyone without the need to log in, and private due to the subject matter. As well, participants could have chosen site seeing it as an intimate space without thoughts for it being potential research material. To that end, the researcher removed identifiers such as aliases, information regarding time and date, and other personal identifiers.
Silvén Hagström 2017 Sweden [48]	Examining online self-disclosure by suicide-bereaved youth, and subsequent reactions to these disclosures	N/A	N/A	N/A	Parents	N/A	Online	Qualitative	Ethical issues surrounding netnography include whether the material is classified as public, private, or a mix of both; in this case, public since the chat could be read and replied to by anyone without the need to log in, and private due to the subject matter. To that end, the researcher removed identifiers such as aliases, information regarding time and date, and other personal identifiers. The material was also translated from Swedish to English; thus, direct quotes cannot be searched online.
Silvén Hagström 2019 Sweden [49]	Examining the differences in how the suicide-bereaved discuss their experience, depending on the social context	N/A	N/A	4 females (interviews) 1 female (theater performance) N/A for online sources	Parents	N/A	Various (including at a theatre and online)	Qualitative	Interviews followed ethical guidelines, and analysis of the theatre performance was shared and approved by the performer. However, there are ethical issues surrounding online material, since it is both public in terms of access but private due to the personal nature of the subject matter. To that end, the researcher removed identifiers. The material was also translated from Swedish to English; thus, direct quotes cannot be searched online.
Søfting, Dyregrov, and Dyregrov 2016 Norway [54]	Children’s experiences participating in death rituals after the loss of a family member	*N* = 22 (11 children, 11 parents)	8–12 (children) N/A for parents	4 boy 7 girls N/A for parents	Parent or sibling	1–3 years	Majority at the participant’s homes	Qualitative	Researchers acknowledged that ethical consideration due to the fact the participants were children in a vulnerable position. Interviews were conducted at the participant’s homes, so that they would be in a familiar environment. As well, the parents were interviewed first so that (1) they could inform the researcher of any considerations that had to be made when the child was interviewed and (2) they would be nearby during the child’s interview in case said child exhibited distress.
Steffen 2017 UK [61]	Exploring sense of presence experiences in a bereaved family	*N* = 4	12–16 (children)	1 female, 2 male (children) 1 female (mother)	Partner/father	~2.5 years	Participants’ home	Qualitative	Potential participants put in contact with past participants in order to hear their experiences and make a more informed decision. Children were assessed regarding whether they were willing or reluctant to participate. Participants provided with information sheets that were tailored to their age.
Thamuku and Daniel 2013 Norway [55]	Exploring the effectiveness of a group therapy program for orphaned children in Botswana	*N* = 18 (sample initially comprised all 44 children at the retreat, narrowed to 10, as their answers were representative for the group; 8 social workers)	13–15	N/A	Parent(s)	N/A	Therapeutic retreat	Qualitative	Had a traditional kgotla (ward) meeting, where verbal consent for the study was given by the Chiefs and the local Social Services office with the children’s guardians present. Guardians and children gave written consent. Professional therapeutic support was available throughout the data collection. Culturally, children are not to question adults and to do so is to be reprimanded. Taking this into consideration, the voluntary nature of participating and the option to withdraw at any point was reiterated throughout the research process. Children and social workers assured of anonymity (no further details given).
Vaswani 2014 UK [62]	Exploring the bereavement experiences and potential relationship with mental health of incarcerated young men	*N* = 33	17.0–20.9	33 males	Parent/primary caregiver, relatives, friends	No explicit range given for the whole group (the “recent” group was bereaved in the past 12 months prior interview)	Young offender institution	Mixed method	Participants were made aware that they could withdraw at any time. All participants were given information on resources they could access while inside the institution. Participants were made aware of the fact any information regarding self-harm/harm to others would be reported to the appropriate bodies. The chaplaincy was involved to address issues and to make referrals if needed. Participants gave consent to be recorded, which were destroyed after transcripts were made. Data are said to have been stored securely (no additional detail given).
Vaswani 2018 UK [63]	Reflection on the research process of a study that examined the loss and bereavement of children in care	*N* = 10	12–17	N/A	N/A	N/A	N/A	Qualitative	Acknowledges that research involving children differs than that of adults since children have a different worldview and different ethical considerations adapting processes geared towards adults would be insufficient. Issues that the researcher encountered included: Gaining ethics approval (there were numerous committees the researcher had to report back to, and an equal amount of feedback that would oftentimes conflict. The power imbalance between the adult gatekeepers and the participating children, as the adults make the decisions and if they refuse then this disenfranchised group even more marginalised, as they take away an opportunity for the children to express themselves. There is also a power imbalance between the researcher (another adult) and the participants. Argued that this imbalance can be addressed by positioning the children as experts. As well, the method of participation can help with the power dynamics; in this case, photo-elicitation engages the children and allows them to control the discussion topics. Balancing the need for a positive relationship with the participants to conduct good qualitative research and not blurring boundaries, which can lead to confused emotions on both the researcher and participant’s parts. Relationships can also affect sense of timing, i.e., having a better sense of whether or not it is appropriate for the subject to participate. Gaining informed consent is questionable due to the study design, gatekeepers, and deciding when children can give consent and not just assent. To that end, there was a grace period after the initial meeting to allow for prospective participants to decide whether they wanted to take part. During qualitative research, participants may make disclosures. Even if the researcher outlines circumstances where disclosures are made and reassures about confidentiality, it does not mean the researcher will forget what they have been told. There is then the dilemma on reflexivity and objectivity as the information may be useful. Distress exhibited by the participants and the role of the researcher in managing it
Weber et al., 2021 Sweden [50]	Examining family communication and mental health of minors whose parent died of cancer	*N* = 62 (23 adolescents, 39 parents)	M = 16.2 (adolescents) M = 48.9 (parents)	15 female, 8 male (adolescents); N/A for parents	Parent	M = 2.9 years (adolescents) M = 2.78 years(parents)	Online/ other (paper versions of the questionnaire)	Quantitative	Specific mention of how “special consideration was taken with regard to the ethical aspects of this study because children and adolescents were involved and the harm a project inflicts must be balanced against the scientific knowledge it produces.” All minors gave assent, and those 15 years and older gave consent, as per Swedish law.
Weber Falk et al., 2022 Sweden [51]	Evaluation of the use of an intervention with families in a palliative homecare setting, with bereavement occurring during/shortly after the intervention	*N* = 24 (14 children, 10 parents)	M = 11.42 (children) M = 48.5 (parents)	5 females, 9 males (children) 5 females, 5 males (parents)	Partner/parent	M = 3.1 years	Therapist’s private practice	Mixed method	On the baseline questionnaire, parents filled out for themselves and their children, and they were given a general overview about the study and were asked to indicate interest/request for more information. Potential participants were contacted via phone to answer questions and gain verbal consent. Written consent and permission to audio record were also collected (although if they declined to be recorded, they could still participate). Participants under the age of 15 gave assent, while those 15 years and older gave consent as per Swedish law.
Wolchik et al., 2006 US [42]	Examining how self-system beliefs impacted stressors and bereaved caregiver-child relationships	*N* = 339 (children) Includes their surviving caregiver	M = 11.46	186 males, 153 females	Parent/parental figure	M = 10.7 months (Time 1 interview)	Participants’ homes, university campus	Quantitative	Recruitment via mail, agencies that would have contact with bereaved children (e.g., schools, hospitals, churches, etc.), and media. Families who did not pass the screening and showed a clinical disorder were given referrals. Caregivers gave consent and minors assent. “Special efforts were used to contact agencies and schools serving ethnic minority families, and barriers to participation were minimized by offering free transportation, babysitting, and dinner for families attending the group program (p224).” Families were paid according to the number of children who took part, once for the interview and another time for the follow-up
Youngblut et al., 2019 US [43]	Exploring children’s’ mental health and school performance after the death of a sibling in the hospital	*N* = 228 (132 children, 96 parents)	6–18 (children) M = 36 (mothers) M = 39 (fathers)	76 females, 56 males (children) 70 females, 26 males (parents)	Child/sibling	Recruitment phase began 4–7 weeks after death	Participants’ homes	Quantitative	Hospital clinical coinvestigators identified potential participants from their facility and provided their contact details which is public information by Florida law. Potential participants also searched for through online obituaries with contact details searched for through databases. Parents contacted to allow them to ask questions about the project and to gauge interest. Collected consent from the parent for themselves and their children, and children under 18 gave assent.
Youngblut and Brooten 2021 US [44]	Exploring the wishes of bereaved children in relation to their deceased sibling	*N* = 132	6–18	58% female	Sibling	Recruitment phase began 4–7 weeks after death	Participants’ homes	Qualitative	Hospital clinical coinvestigators identified potential participants from their facility and provided their contact details which is public information by Florida law. Potential participants also searched for through online obituaries with contact details searched for through databases. Parents contacted to allow them to ask questions about the project and to gauge interest. Collected consent from the parent for themselves and their children, and children under 18 gave assent.

**Table A3:** Summary of grey literature.

Author, Year Submitted, Study Origin	Study Purpose	Sample Size	Age	Male/Female	Deceased’s Relation to the Participant	Time Since Bereavement	Setting	Study Design	Ethical Issues
Barrett 2003 US [70]	Examining the perceptions of participants at a bereavement camp	*N* = 10 (children) *N* = 67 (parents)	8–17	N/A	Family member	N/A	N/A	Qualitative	Obtained written informed consent (does not specify from who), also obtained permission to tape, record, and transcribe. Provided pseudonyms. Discusses the need for participants to be provided all relevant information in order to make informed decisions vs. the reality that qualitative interviews can be unpredictable, even with a script and prompts—can be addressed by allowing participants to stop the interview/not answer questions they find difficult. Discusses the advancement from making sure researchers do no harm to seeking ways for the research process to be mutually beneficial for the researcher and participants. Researcher draws distinction between research and therapy, asserting that the project is on the former and not the latter. Expands on the view that social science research should be collaborative and participatory. Raises the question of whether human research ethics policies are in place to protect participants or the institutions associated with the research.
Baxter 2019 UK [88]	Examining children’s and young people’s educational experience after parental bereavement (via suicide)	*N* = 3	15–20	2 males 1 female	Parent	1–3 years	Charity	Qualitative	Researcher acknowledged that the participants were vulnerable in part due to age and the research topic, as well as the power dynamics between the adolescent and their support worker/deceased parent. To address this imbalance, youth were explicitly told that they could withdraw from the study at their discretion. Researcher underwent a criminal check before working with the participants. Did not recruit participants directly, but rather sent the relevant information to the charity which acted as an intermediary. After initial contact, consent was obtained (from the participant/participant’s guardian). Pseudonyms were utilised and the interviews were coded. Researcher placed importance in obtaining consent throughout the study. In order to reduce anxiety, the researcher took time to connect with the participants through an activity during the introductory meeting. This also gave the participants a chance after the meeting to decide if they wished to proceed in the study. As well, during the study the participants had the option of having their support worker present, and could receive help from the afterwards if needed. The researcher provided information regarding available support resources and checked in on the participants via email throughout the process. Contingency measures were also placed in case a participant experienced distress. Dilemma of disclosing own relevant experience to participants (risk–benefit analysis of whether it would influence their recount or if they would feel deceived). Researcher ultimately decided to disclose after the interviews. Considerations for researcher self-care, as it is a sensitive topic and her well-being would also impact the participants. This took the form of support from her research supervisor and access to support agencies.
DeSantis 2011 US [71]	Exploring aspects of the adolescent bereavement process, i.e., their experiences and characteristics that allow them to adapt	*N* = 20	13–18	11 males 9 females	Guardian, friend, sibling, or ‘other’ (unspecified)	Less than 6 months–over two years	Online	Quantitative	Did not directly recruit, but rather through intermediaries. Provided assent and consent forms when advertising for participants. Used email address in lieu of names as initial identification, which was then replaced with a number after the data were received. The data were stored on a hard drive in a secure location. Participants were given feedback on the VIA-IS score, and a chance to win a gift card.
Dickerson 2011 US [72]	Exploring the perceptions of adolescent parents on the cause(s) of their perinatal loss	*N* = 8	17–18	7 females, 1 male	Child	Less than a year–approx. 2 years	Telephone/in-person (location?)	Qualitative	Support agency for bereaved adolescent parents acted as gatekeepers, only granting access to clients after IRB approval. Bereavement coordinator facilitated introductory meetings with potential participants. The coordinator was the one to ask clients if they wished to participate in the study. Consent form for guardians, assent form for participants. Participants told it was voluntary and that if they did take part, they could also choose to withdraw at any time. Participants and their guardians were made aware of potential risks (psychological in this case). Minimized risk by informing participants that their participation was voluntary and that they could withdraw from the study at any time with no repercussions. Had referral for professional help should participants seek it. Data were coded to protect confidentiality, and pseudonyms were used. Transcripts stored electronically and in paper format locked in the researcher’s office. When prerequisite time has passed, researcher would delete data and sanitize disk to ensure all data are gone. Participants signed consent forms (as they were all 18 years of age but one). Discusses the power dynamics between adolescents and adults (parents and researchers) and whether they can make an autonomous decision or bow to authority. To counteract this the researcher took several steps, including: asking the parents not to influence the adolescent’s decision making; providing notification to parents to provide proof the adolescent’s well-being is being safeguarded; and having the parents present since the researcher was a stranger to the adolescent. In regard to the participant under the age of 18, the researcher gained her assent in addition to parental consent to reinforce the belief her consent was important and to make her feel her participation was important to the study. The researcher expressed a belief in gathering background knowledge of the participants to better approach them as individuals and lessen any sense of unease the research setting may cause. To this end, the researcher talked with the participants’ parents and informally met the participants before the interview. Researcher acknowledged role as a social worker and how as a member of the National Association of Social Workers must adhere to the group’s Code of Ethics.
Doran 2001 US [72]	Exploring the bereavement experience of Mexican American families who have lost a child	*N* = 12 (9 family members; 3 ancillary contacts)	8–72 (family members)	7 females, 2 males (family members of deceased) 1 male, 2 female (ancillary contacts)	Child/sibling; other	Min. 2 years	N/A	Qualitative	Used personal contacts to reach out to gatekeepers in the Mexican American community for assistance in finding participants for the study. Respected wishes of potential participants—if the nominated individual declined to be interviewed/did not phone back, this was understood to be an unwillingness to participate. Discusses the issue of maintaining anonymity when conducting group interviews; researcher made efforts to preserve anonymity in regard to individual interviews by not sharing information between family members. As well, participants were given pseudonyms and nonessential data and parts of the stories were changed to further disguise identity. Acknowledges risks inherent in the study stemming from the fact that the topic may evoke emotional pain. To lessen the risk, participants had the option of changing their participation or ceasing to take part at all. As well, referral for professional help that would also be aware of cultural sensitivities was available if necessary. Specific safeguards for child participants were put in place, and included: having children aged 6–12 draw and reflecting upon their drawings when possible in lieu of the interview; adolescents aged 13–17 were invited to be interviewed, with the knowledge that parents had the right to any information their children shared. Parents were asked to allow for confidentiality to be applied to child–researcher interactions (excluding information of harm, either self-inflicted or by others, in which case parents would be made aware). Could choose what language the interview would be conducted in according to their comfort. Will hold data for a specified period of time in a secure location, after which it will be erased/shredded.
Duke 2013 US [74]	Examining the impact of a bereavement camp on participants’ hope, depression, and self-perception	*N* = 160	9–17	91 females, 69 males	Family member, other	Within the last 12 months–over 5 years (from camp attendance)	Camp, participants’ homes	Quantitative	National program manager served as intermediary in gauging interest among camps regarding study participation, and distributed advertisement. Mentions the issue of data sharing across borders—for this study, approval to conduct the study with the Toronto camp hinged on a Data Sharing Agreement in which identifying information would not be sent across the border. Gathered parent consent and youth assent. Provided a study overview with the consent forms. 2011 response levels were low, so in 2012 greater incentive was provided for the campers to complete the assessments. In this case, the incentive took form of entry to a raffle for a donated gaming system. The information was given in the initial study overview and campers were also told of their chances of winning. Campers and their guardians told their information would be confidential and that that participation was voluntary and that even if they participated, they could later withdraw if they so wished. Also told who to contact if they had any questions. The camp clinical director was the one to introduce the study to potential participants and was also the one to gather consent/assent.
Edelschick 2005 US [75]	Examine the effects of bereavement on high school GPA and self-competence as perceived by the bereaved adolescents	*N* = 255 (34 were bereaved)	Grades 9–12	40.7% males, 59.3% females (out of 255 participants)	Family member who lived in the same household as the participant for a min. of 2 years	Loss from as young the age of 2	School, at bereaved participant’s homes	Quantitative	Acknowledged there is bias inherent in the research design, e.g., what questions and participants are included in a survey Risk–benefit analysis of conducting the study. Administered the surveys alongside two social workers and kept a lookout for any signs of participant distress. Informed the participants that if they required assistance, the social workers were available, and contact information for both the social workers and the researcher was included on the consent form. Personally thanked and checked upon each bereaved participant. Gave participants small tokens of appreciation (donuts and gift cards). Open-ended question included in the survey that participants could use to ask for help, tell a secret, or convey their emotional distress.
Godder 2008 US [76]	Examining psychological symptoms of traumatically and non-traumatically bereaved children	*N* = 83	8–12	49 females 34 males	Parent (traumatically bereaved), relatives or close adult figure (non-traumatically bereaved)	Varied	Participant’s home (traumatically bereaved) School (non-traumatically bereaved)	Mixed methods	Traumatically bereaved: participant’s guardians and participants provided consent and assent, respectively. The families were made aware of what the evaluation entailed, and that they could withdraw at any point in time. Clinicians made sure that participants understood the confidential nature of the evaluation but also the limitations of said confidentiality. Children receive assessment. Non-traumatically bereaved: researcher read a script to the potential participants outlining what the research entails and eligibility criteria. The children were then given an information letter to take to their guardians, along with permission forms. Children also had to give verbal assent. Signed permission forms could be dropped off in a box at a secure location in the school. Upon the start of participation, participants’ names were swapped with identifying numbers instead. The interviews took place in a quiet location to ensure privacy. Two participants expressed thoughts of self-harm, and the interviewer performed a risk assessment. After finding them to not be in immediate danger, they once again explained the limitations of the confidentiality agreement and spoke to their parents about possible resources for the children. As well, the researcher’s committee member, who is a licensed clinical psychologist, was consulted. Both groups received a gift card for their participation.
Greidanus 2005 Canada [89]	Narrative inquiry into the experiences of bereaved children	*N* = 16 (children) 3 mothers acted as informants in the longer narratives included in the study	5–12	9 boys 7 girls 3 females (mothers)	Relative	varied	Art-focused grief support group; various other locations	Qualitative	Narrative inquiry makes participants into collaborators, leading to a deeper relationship between the researcher and participant than is norm. States that the research was conducted following the ethical principles of “respect for human dignity” and “respect for free and informed consent” (p. 50) as outlined in the college’s ethical guidelines. Provided information to participants so that they could make an informed decision. Told that they could withdraw from the study at any point Gained consent from the children themselves. Acknowledged additional ethical considerations arise when conducting research with minors (“respect for vulnerable persons,” p. 51). Participants are doubly vulnerable as children experiencing a distressing event. Participants were told they can stop the interview at any time/not answer questions. Acknowledged possible conflict due to dual roles of researcher and bereavement counsellor and had resources to outside support available for participants. Informed participants of steps taken to ensure privacy (e.g., pseudonyms); possible benefits/risks. Ensured the study was within the researcher’s abilities to carry out.
Hay 2008 US [77]	Exploring how social support received by caretakers in a bereaved families impacts their dependent children	*N*= 40 (20 mothers, 20 children)	7–14 (children) 23–55 (mothers)	20 females (adult) 3 males (adult); excluded from data analysis 10 girls 10 boys	Family member	Less than 6 months–less than 4 years	Participant’s homes (mailed the research packets, which included the questionnaires)	Quantitative	Acknowledged that due to the emotional vulnerability associated with grieving, there are ethical issues surrounding research with this population and random sampling may be detrimental to their well-being. In addition, acknowledged that bereaved children are “an even more potentially vulnerable and protected population (p. 89).” There is also the issue with study design, in that if a control group is included, the participants would not receive necessary treatment, which is unethical. Found literacy prevented participation of certain families, “primarily of African-American background (p. 91).” Even with the offer of accommodations, these families refused to participate. This leads to an underrepresentation of the minorities who participate in the bereavement camp.
Hirschson 2013 South Africa [92]	Examining the use of art therapy for AIDS-bereaved orphans in South Africa	*N* = 16	~11–14, ages for 8 participants unknown	9 males, 7 females	Parents, other family members	Up to 12 years from date of interview	Children’s home	Qualitative	Acknowledged dependability is subject to the limits of being able to replicate therapy sessions. Had issues in regard to data storage, as there were large amounts and of different mediums coupled with the fact that the products would be returned to the participants if they so wished (to be determined through a discussion with said adolescents). There was also the matter of respecting the participants’ engagement with the program as well as their produced works—this respect was demonstrated by abiding participant decision on whether to share their creation or not. Acknowledges that specific guidelines regarding respect were in place, owing to the fact that the participants were impacted by the HIV epidemic. This was done not only for the sake of the participants, but also to aid in societal betterment. Motivation to find ways to help minors find ways to live healthier along with belief that participants be empowered in understanding their grief lead to the decision to use art therapy. Discusses what the researcher believes to have been a main ethical issue for this study, which was that based on earlier research, the study was based on the premise the bereaved subjects displayed aggression due to their loss. This aggression meant that the proposed use of dance therapy was acceptable. However, it was found that this premise was wrong throughout the research process, and as different forms of art therapy was being incorporated into the study, limiting it to dance would be wrong. Preservation of confidentiality and anonymity in order to maintain good relations with and between the participants. Upheld social justice, as it is crucial to participation/collaboration by creating opportunities for participants to share their thoughts and feelings in a safe environment. Researcher also heeded cultural differences in demonstrating grief. Gained consent from the management at the children’s home (in lieu of a parent/guardian) and assent from the adolescents who were informed of the purpose of the study. Social worker volunteered to speak with the participants before the first session in order to explain the study, with a script she could translate to the languages spoken by the adolescents. As well, during the study, the researcher routinely gauged the adolescents’ understanding via verbal and nonverbal means. Researcher made arrangements in case there was a need for professional referral. Discusses the hypothetical dilemma of funding/sponsorship and how it can impact aspects of the research such as the number of intervention sessions that can take place (circumvented in this study since there was no cost for the children’s homes or any funders). This in turn can make it so that the potential risks outweigh the benefits for participants. Acknowledges the dilemma of participants giving information to researchers that could lead to them being harm if shared with others. Discusses the rights of children, specifically autonomy (whereby the participants were allowed to express themselves in the manner that befitted them) and beneficence (researcher considered the possible risks vs. benefits for participants, and took steps to minimize risks).
Horsley 2003 US [78]	Examining the effectiveness of the parent guidance intervention in communication between bereaved parents and their children	*N* = 5 (adolescents) Parents were also involved as well	14–17	2 females, 3 males (adolescents)	Child/sibling	1–2 months from study date	Participants’ homes	Mixed methods	Participants were contacted weekly during baseline to address any questions they might have and to ensure compliance. Potential participants were contacted by the chapter leaders; data were coded and secured in a locked cabinet. Explicitly stated in the inclusion criteria that participation must be voluntary. Researcher adhered to the ethical guidelines of the American Psychological Association (1995) for the protection of the participants’ rights—potential participants informed of what the study entails, assured of confidentiality and anonymity, and that they could withdraw at their discretion at any time, told the results would be available at the end of the study. Gained parent and adolescent consent. Sessions were free, and subjects were provided compensation twice, for completion of the assessment and treatments, respectively. Parents were also compensated for their time. Participants who asked for additional therapy at the end of the study were given referrals.
Lai 2013 US [79]	Exploring Hong Kong children’s expressions of grief following the death of their parent due to illness	*N* = 21 (children, N/A for adults)	3–7 (N/A for adults)	10 males 11 females (N/A for adults)	Parent	1 year or less	Child and family bereavement centre in Tuen Mun Hospital, Hong Kong	Qualitative	Guardians were informed about the research and what taking part in it entails and were reassured that participants could withdraw at any given time. Researcher collected signed consent and assent forms. Pseudonyms were utilised to maintain participant privacy. Participants were assured their data would remain confidential, except in the case when it was determined there was harm involved. Confidentiality had to be breached in the case of one participant, with the permission of her mother, in order to refer the girl to a professional. This was due to sexual play being exhibited during the play session. At that point the participant would be referred to the relevant authorities/help. Collected drawings would be destroyed 5 years after the study was published. Interviews were conducted in Cantonese as that was the native language of both the researcher and the participants.
Leigh 2016 US [80]	Examining the impact of legacy building interventions on bereaved families	*N* = 16 (11 parents, 5 siblings)	6–13 (siblings)	10 females 6 males (2 females 3 males adolescents)	Child/sibling	Within the last 10 years	Hospitals/hospices/palliative care facilities or over the phone	qualitative	Participants were required to self-determine whether they were mentally healthy to participate, discussing the matter with trusted others and parents providing insight into the well-being of their children. Safety and confidentiality listed as priorities. Explicitly states that one concern was obtaining uncoerced/influenced assent from the children. Gained consent from the parent and child. Paediatric palliative care clinician offered her services in identifying potential participants. Participants told that they could withdraw from the study at any point.
Lundberg 2019 Sweden [91]	Examining the psychological well-being of bereaved families, with a focus on young adults	*N* = 25 (study 1) N = 77	Over 18 (study 1) 16–28 (studies 2–4)	12 females 12 males (study 1) 64 females 13 males (studies 2–4)	Family members (study 1) Parents (study 2–4)	Approx. 6–9 months (study 1) Min. 2 months from study date (study 2–4)	Palliative care service (study 1–4)	Mixed methods	Acknowledges the fact that the study topic may give rise to difficult feelings, but at the same time, participants may gain satisfaction in receiving attention for their experiences and in contributing. Study adhered to the Declaration of Helsinki and research ethics principles set by The Swedish Research Council (information, consent, confidentiality, utilization). Participants in studies 2–4 were able to participate in the support groups even if they did not take part in the study. In study 1, participants were given an information letter and provided consent. Were explicitly told none of the interviewers worked at the palliative care service, in order to reassure them they could speak freely. The interviewers also had experience meeting bereaved families, and as such, provided information on support services when needed. In studies 2–4, participants were told about the confidentiality measures (regarding group leaders and the fact that the researchers were uninvolved with the support groups). Consent was implicitly given by completion of the questionnaires, which were screened for signs of poor mental health. Those with certain scores were subject to additional screening, and if the result was at a certain threshold, the participant would be contacted for assessment and an offer of additional support. Potential participants told they could withdraw at their discretion, and those in studies 2–4 were told that they could participate in the support groups regardless of if they took part in the study. Participant anonymity and confidentiality was ensured, and data coded (identification data stored in a secure location).
McQuaid 2005 US [81]	Examining the effects of age, gender, family functioning, and peer support on children bereaved by the attack on 9/11	*N* = 79 (children) Also includes their caregivers (exact number unknown)	8–18	4% female, 57% male (children)	Father, friend, other	Post 9/11	NYU Child Study Center offices, Fire Department Counseling unit offices, participants’ homes	Quantitative	Database of potential participants from The Silver Shield Foundation. Wanted to make the program as accessible as possible; thus, established relationships with the fire department mental health units. Gained consent and assent from caregivers and children. Participants were compensated for their time.
Mohan Van Heerden 2002 Canada [90]	Exploring the use of art therapy with bereaved children	*N* = 5 (children); surviving parents also interviewed to gather additional family information	9–12	5 females (children)	Parent, other family member	1–4 years	N/A	Qualitative	Children given the option to choose their own pseudonyms, although some (along with one parent) declined to do so. When discussing confidentiality with the parent, he brings up the point that part of the research’s purpose is to make death and associated topics in relation to children more open and that the event is not something to hide, but rather one that would hopefully help others in a similar situation. Consent gained from parents and the children who participated, and additional permission sought from participants and the palliative to include the children’s works in the thesis. Discusses ethics musical therapists are beholden to.
Munholland 2000 US [82]	Exploring the experience of parental bereavement in adolescence	*N* = 15	13–19	8 girls 7 boys	Parents	7–30 months prior to participation	The Dougy Center, participants’ homes	Qualitative	Study design allows for participants to voice their experiences. More sensitive information can be disclosed in a private, individual setting rather than in a group setting. By centring the interview on the participant’s experiences, they have some semblance of power as they are controlling the flow of the discussion. Did not include one teen group, as the researcher also acted as a facilitator for their caregivers-in this way, avoid accidental coercion, fears of confidentiality being breached (either on the caregiver or participant’s sides).
Naidoo 2014 South Africa [93]	Examining the support provided by a school-based team geared towards orphans	*N* = 10 (6 children 4 caretakers)	N/A	N/A	Parents	N/A	Primary school	Qualitative	Acknowledges the fact that qualitative research can cross into the private aspects of participants’ lives, and they may feel it to be intrusive/embarrassing. To counteract this the researcher assured participants their privacy was of utmost importance. Letters of consent were sent to the foster parents and the orphans, among others. Took into account that participants may have poor reading skills so before each interview participants were given a verbal overview of the study and allowed to ask questions. The legal guardians and the children gave consent. Acknowledges that orphans are a vulnerable population thus screened them before participation in the study. Legal guardianship was confirmed through proof of court order as well as the foster care grant provided by the Department of Social Development. Participants were made aware about the research and were told that confidentiality was a priority. Before giving written consent, it was made clear that participants would not be financially compensated for their time (although their transportation would be covered). Discussion about the importance of informed consent, and how participants should not be deceived—to this end, they debriefed participants before the interview. Discussion surrounding privacy and confidentiality: Among the steps taken was the use of pseudonyms. Cites potential embarrassment as one reason as to why privacy is important. The ethics of accurate data—researcher reinterviewed select participants to gather more detailed descriptions from them.
Osemwegie 2010 US [83]	Exploring the experiences of Nigerian children bereaved due to a stigmatised death	*N* = 16	3–10	9 boys 7 girls	Parent, family member, neighbour	Within 3 years of participation	Participants’ homes, out of home venue (e.g., school, church)	Qualitative	Researcher had to obtain a clearance certificate from the National Health Research Ethics Committee of the University of Benin Teaching Hospital to conduct the study in Nigeria. In addition, had to receive permission to engage a local mentor due to the study topic and participant group, as per the requirements of the Nigerian National Health Research Ethics Committee. All quotations are verbatim. Children gain an outlet to discuss their experiences vs. emotional distress they may feel during the process. Written consent obtained from the guardians. Assent from children aged 7 years and older was gathered as well. It was made clear to participants that they could withdraw from the study at any point in time, and were questioned to test their understanding and encouraged to ask questions in turn. Had referrals to a clinical psychologist available for any participant who was distressed during the research. Clinical materials were selected with consideration to cultural values. The children’s identities were only revealed to those whose permission was needed to conduct the participant observation. Pseudonyms and numbers were used as placeholders, and only the researcher could link the names and pseudonyms together. Both hardcopies and electronic information was secured, and information that was no longer necessary was destroyed with a witness on hand. Research assistants had to sign a confidentiality agreement. Participants were informed that all information would be kept confidential, except for claims of abuse, which would then be forwarded to the appropriate authorities. Of special consideration was due to the nature of the deaths, legal matters would be involved. As such, information was coded.
Ross 2000 US [84]	Exploring children’s perceptions on the support they received following parental bereavement	*N* = 22 (11 children, 11 parents)	10–16	9 boys 2 girls 8 mothers 3 fathers	Parent	1–5 years	Participants’ homes (questionnaire)	Mixed methods	Researcher initially reached out via telephone and discussed the study with the parent and child. After obtaining agreement to participate, the parents were sent a consent form covering both their child and their own participation. Children were given a form detailing information about the study, which they reviewed with the researcher. There was also a section on the form for consent, and their signed consent was collected. Acknowledged the implications of the researcher–participant relationship, and that the impact the study can have on the participant is the researcher’s responsibility to address if there is a possibility of the children being retraumatized. The researcher looked at available evidence and contacted leading researchers in the field for their opinion. Found that there is no evidence of such risk. Verbally acknowledge the participant’s loss and validate their experiences. Researcher had resources available should the participants like support. Verbally assured the participants on the steps taken to keep their information private, utilised pseudonyms. As well, the interviews were personally transcribed by the researcher.
Sirrine 2013 US [85]	Examining the continuing relationship bereaved youth and their caretakers have with the deceased	*N* = 96 (50 youth, 46 caregivers)	11–17 (youth) Mean = 45.17 (median = 46, SD = 10.95) (caregivers)	52% male (youth) 48% female (youth) 84.8% (female caregivers)	Family members	1 month–6 and a half years	Bereavement centres	Mixed methods	The researcher had a training session for the assistants who were involved in administering the measures and collecting data. They were also briefed on topics such as consent, confidentiality, and research protocol. Written consent and assent from the caretakers and youth aged 12–17 was collected. Verbal assent was obtained from those aged 11. The researcher did not collect assent from participants who were a part of the support group she facilitates. The research material was stored in a secure location (for both electronic and hardcopies). Gift card awarded for participation.
Wehmeyer 2011 South Africa [94]	Exploring posttraumatic growth in adolescents bereaved of both parents	*N* = 4	13–15	3 females, 1 males	Parents	Minimum of 2 years before study	Children’s home	Qualitative	Granted permission by the social worker to conduct the study at the children’s home, and worked in tandem with said social worker in selecting participants. Social worker signed the consent forms in the capacity of legal guardian. Researcher then met with the participants to explain the study, and to give them the consent forms. Location for the interviews were chosen based on participants’ comfort. Researcher acknowledges that study is on a sensitive topic, and extra caution must be given because participants are adolescents. Justifies the use of qualitative methods as a means of ensuring participants are treated with care and empathy, and to account for subjectivity regarding their experiences. Researcher acknowledged informed consent to be one of the most important ethical issues for them. Mentions the dignity and welfare of participants, steps taken to ensure confidentiality. Acknowledges that in keeping with ethics, results must be reported accurately, and fraud/plagiarism is to be avoided.
Wheat 2011 US [86]	Explore the bereavement experiences of adolescents that are part of an ethnic minority	*N* = 3	15–16	3 females	Peer	Within 2 years of participation	Participant’s home	Qualitative	Recruited via adult gatekeepers rather than directly contacting the potential participants. Used pseudonyms and stored the data in a locked file. Planned to destroy identifying information when the study was completed. Other electronic and paper copies with pseudonyms and descriptions are planned to be destroyed after a year. The researcher did not save participant’s contact information during the study. Transcriptionists were made to sign a confidentiality statement. The peer reviewer gave verbal confirmation to maintain confidentiality. Gained written assent and consent from the participant and their guardian, respectively, with the forms adapted for the cognitive levels of each signee. No deadline to minimise pressure. Offered a small incentive upon agreement to avoid exploitation. Monitored participant’s emotional state during the interviews and provided access to resources for counselling.
Zvokel 2007 US [87]	Examines the role of literature in bereaved children processing the change in their identity due to their loss	*N* = 11 (children) Program staff and parents also provided information	3.5–6 years old	5 females, 6 males	Parents, siblings, other relatives	~3 months-4 years	Hospice centre	Qualitative	Gained consent from the parents, who understood that participation was voluntary. There was no anticipated risks and the children could withdraw at any point. Gained consent from the hospice network and the social workers in the bereavement groups. Anonymity was assured, but one participant was not given a pseudonym with permission from her mother due to the nature of her creation. However, the consent to use her actual name only extended to the dissertation. Ethics of gaining private information during debriefing sessions in order to have better context—researcher decided this was not part of the study scope and thus not covered by the parents’ consent.
